# Supplementary material for: Socio-Demographic and Knowledge-Related Determinants of Vitamin D Supplementation in the Context of the COVID-19 Pandemic: Assessment of an Educational Intervention
Source: Front Nutr. 2021 Jun 2;8:648450. doi: 10.3389/fnut.2021.648450 (PMC8206500; doi:10.3389/fnut.2021.648450)
Supplement: Supplementary file 1 [file Table_1.DOCX]

**SF 1.** Vitamin D-related knowledge questionnaire: Questions and choice responses (English translation)

| **Questions and choice responces** | |  |
| --- | --- | --- |
| **Q1** | **Where do you think vitamin D comes from? (check all that are correct)** | |
|  | ***Internal note on dimension: Food and other vitamin D sources*** |  |
| A | Don't know |  |
| B | Fruits |  |
| C | Water |  |
| D | Vegetables |  |
| E | Fatty fish* |  |
| F | Vitamin D supplements* |  |
| G | Sun* |  |
| H | Air |  |
| I | Select cereals* |  |
| J | Milk/ Dairy* |  |
| K | Nuts |  |
| L | Cod liver oil* |  |
| M | Chicken |  |
| N | Eggs* |  |
| **Q2** | **Vitamin D helps with which of the following health effects (check all that apply):** | |
|  | ***Internal note on dimension: Health impact*** |  |
| A | Don't know |  |
| B | Bone health* |  |
| C | Immune health* |  |
| D | Prevention of Rickets* |  |
| E | Vision Health |  |
| F | Pregnancy/ Breastfeeding* |  |
| G | Hair Growth |  |
| H | Prevention of diabetes* |  |
| I | Cardio-vascular health* |  |
| J | Cognitive health* |  |
| K | Cancer prevention* |  |
| L | Osteoporosis prevention* |  |
| M | Disease prevention* |  |
| N | Skin softness |  |
| O | Calcium absorption* |  |
| P | None of the above |  |
| **Q3** | **What is the daily amount of vitamin D currently recommended for adults?** | |
|  | ***Internal note on dimension: Dietary needs*** |  |
| A | Don't know |  |
| B | 200 I.U. (International units) - 5 µg |  |
| C | 400 I.U. - (10 micrograms) |  |
| D | 600 I.U. - 15 µg* |  |
| E | 800 I.U. - 20 µg* |  |
| F | 1000 I.U. - 25 µg |  |
| G | 1500 I.U. - 37,5 µg |  |
| H | 2000 I.U. - 50 µg or more |  |
| Q4 | **How much time would the average fair-skinned person need to spend in the sun to get enough vitamin D, if their bare legs and arms were exposed?** | |
|  | ***Internal note on dimension:*** Sun exposure and biosynthesis |  |
| A | Don't know |  |
| B | Less than 10 minutes per week |  |
| C | 10-60 minutes per week* |  |
| D | 1-2 hours per week |  |
| E | More that two hours a week |  |
| Q5 | **Factors that can decrease the amount of vitamin D a person can get are (check all that apply):** | |
|  | ***Internal note on dimension: Other factors and biosinthesis*** |  |
| A | Skin pigment* |  |
| B | Shade/ clouds* |  |
| C | Time of day* |  |
| D | Latitude* |  |
| E | Season* |  |
| F | Age* |  |
| G | Pregnancy/ lactation* |  |
| H | Fatty diets |  |
| I | Sunscreen use* |  |
| J | Vegan diet* |  |
| K | Vegetarian diets* |  |
| L | Lactose intolerance* |  |
| M | Dairy allergy* |  |
| N | Pollution* |  |
| O | Wind |  |
| P | Smoking |  |
| Q | Body Mass Index* |  |
| Q6 | **What percentage of the Slovenian population is estimated to be vitamin D insufficient (i.e., getting less than what is recommended)?** | |
|  | ***Internal note on dimension: Defficiency prevalence*** |  |
| A | 0% |  |
| B | 0,5% |  |
| C | 5% |  |
| D | 10% |  |
| E | 25% or more* |  |

**Note:** (*) Represent the correct choice options
